# Effects of Dietary Fruit and Vegetable Consumption on Prediabetes: A Systematic Review and Meta-Analysis

**DOI:** 10.3390/nu18091391

**Published:** 2026-04-28

**Authors:** Seung-Hee Hong, Yun-Jung Bae

**Affiliations:** 1 Department of Food and Nutritional Science, Shinhan University, Uijeongbu 11644, Gyeonggi, Republic of Korea; 2 Food and Nutrition, Division of Food Science and Biotechnology, Korea National University of Transportation, Jeungpyeong 27909, Chungcheong, Republic of Korea

**Keywords:** prediabetes, vegetable, fruit, consumption, meta-analysis

## Abstract

**Background/Objectives**: Previous studies have reported inconsistent findings regarding the association between fruit and vegetable consumption and prediabetes. Therefore, we aimed to investigate this association using a meta-analysis, following the Preferred Reporting Items for Systematic Reviews and Meta-Analyses (PRISMA) guidelines. **Methods**: Relevant studies were identified through systematic searches of PubMed, EMBASE, and the Cochrane Library through January 2026 using predefined keywords. Pooled odds ratios (ORs) with 95% confidence intervals (CIs) were estimated using a random-effects model. **Results**: A total of 20 observational studies were included, comprising 16 cross-sectional studies, one case–control study, and three cohort studies, with 24,660 cases of prediabetes among 182,387 participants. Higher fruit and vegetable consumption was significantly associated with a lower risk of prediabetes (OR, 0.82; 95% CI, 0.74–0.91; *I*^2^ = 91.9%). Subgroup analysis by study design showed a significant association in cross-sectional studies (OR, 0.81; 95% CI, 0.70–0.94; *I*^2^ = 91.6%; n = 16), whereas cohort studies showed no significant association (OR, 0.97; 95% CI, 0.92–1.02; *I*^2^ = 15.1%; n = 3). Subgroup analyses by sex indicated a significant inverse association among male participants (OR, 0.77; 95% CI, 0.61–0.97; *I*^2^ = 63.3%; n = 4), but not among female participants (OR, 0.97; 95% CI, 0.69–1.37; *I*^2^ = 82.1%; n = 3). **Conclusions**: Overall, this meta-analysis indicates that greater fruit and vegetable consumption is associated with a reduced risk of prediabetes. However, additional prospective cohort studies are required to clarify potential confounding factors and confirm this association.

## 1. Introduction

Diabetes is a chronic disease with a steadily increasing global prevalence [1,2] and represents a major public health concern. The condition is associated with multiple complications, including cardiovascular disease, kidney disease, and neuropathy, which substantially reduce quality of life and increase socioeconomic burden [3,4,5]. Both genetic predisposition and lifestyle factors contribute to the development and progression of diabetes, with dietary factors recognized as key modifiable determinants for prevention and management [6,7]. Diet quality and the composition of food groups influence glycemic control, insulin resistance, body weight regulation, and inflammatory responses, thereby affecting the underlying pathophysiology of diabetes [8,9]. Epidemiological studies and meta-analyses indicate that consumption of several food groups—including whole grains, nuts, dairy products, fish, fruits, and vegetables—is significantly associated with the risk of type 2 diabetes, disease management outcomes, and overall mortality, including diabetes-related mortality [10,11].

Fruits and vegetables are important sources of vitamins, minerals, dietary fiber, and diverse phytochemicals, including polyphenols. Consumption of these foods has been reported to improve insulin sensitivity and support glycemic homeostasis through multiple mechanisms, including body weight regulation, reduced inflammation, decreased oxidative stress, and modulation of the gut microbiota. These mechanisms are considered important in the development and progression of diabetes [12,13].

However, the association between fruit and vegetable consumption and diabetes may depend not only on the quantity consumed but also on consumption patterns. For example, metabolic effects differ according to whether fruits are consumed as whole fruits, juices, or processed products. Similarly, the health effects of vegetables may vary depending on preparation methods, such as raw vegetables, cooked vegetables, pickled vegetables, or vegetables consumed with high-calorie dressings [14,15]. Previous studies have reported inconsistent findings; fruit juice consumption has been associated with an increased risk of type 2 diabetes, whereas whole fruit consumption has been associated with reduced risk [16]. These differences highlight the need for further investigation of fruit and vegetable consumption in relation to diabetes risk.

Prediabetes is an intermediate metabolic state between normal glycemic status and diabetes and is characterized by an elevated risk of progression to diabetes [17]. This stage represents a critical window for intervention because lifestyle modification can prevent or delay disease onset. Dietary and behavioral strategies that reduce insulin resistance may enable individuals with prediabetes to return to normoglycemia [17,18].

Nevertheless, most studies examining fruit and vegetable consumption have focused primarily on patients with type 2 diabetes. Evidence regarding the association between fruit and vegetable consumption and prediabetes remains limited. In particular, systematic reviews and meta-analyses evaluating this relationship have not been sufficiently reported.

Therefore, the present study conducted a systematic review and meta-analysis to evaluate the association between fruit and vegetable consumption and prediabetes. By synthesizing and quantitatively summarizing existing evidence, this study aims to clarify the potential role of fruit and vegetable consumption in prediabetes and provide evidence to inform dietary recommendations and public health strategies for diabetes prevention.

## 2. Materials and Methods

### 2.1. Search Strategy

We conducted a systematic review and meta-analysis of observational studies examining prediabetes. This review followed the Preferred Reporting Items for Systematic Reviews and Meta-Analyses (PRISMA) guidelines, and a checklist was completed [19]. PubMed, EMBASE, and the Cochrane Library were systematically searched for eligible studies published through January 2026. Medical Subject Headings (MeSH) terms were used for PubMed, together with keywords related to fruit and vegetable consumption and prediabetes.

The PubMed search strategy was as follows: (“prediabetic state” [MeSH Terms] OR (“prediabetic states” [Text Word] OR “prediabetes” [Text Word] OR “impaired fasting glucose”[Text Word] OR “impaired glucose tolerance” [Text Word] OR “hemoglobin A1c” [Text Word])) AND (“Vegetables” [MeSH Terms] OR “Fruit” [MeSH Terms] OR “Citrus” [MeSH Terms] OR (“vegetable” [Text Word] OR “Vegetables” [Text Word] OR “Fruit” [Text Word] OR “fruits” [Text Word] OR “plant capsule”[Text Word] OR “capsule plant” [Text Word] OR “capsules plant” [Text Word] OR “plant capsules” [Text Word] OR “plant arils”[Text Word] OR “berries” [Text Word] OR “berry” [Text Word] OR “legume pod” [Text Word] OR “legume pods” [Text Word] OR “pod legume” [Text Word] OR “Citrus” [Text Word] OR “citrus fruits” [Text Word] OR “cruciferae” [Text Word] OR “cruciferous vegetables” [Text Word] OR “mediterranean diet” [Text Word])).

### 2.2. Eligibility Criteria and Study Selection

Two authors independently screened all studies identified from the databases and reference lists. Disagreements during the selection process were resolved through discussion. The inclusion criteria were: (1) observational studies (cross-sectional, case–control, cohort); (2) fruit and vegetable consumption as the exposure of interest; (3) reported associations with prediabetes expressed as odds ratios (ORs) with 95% confidence intervals (CIs); (4) studies conducted in the general population; and (5) publication in English.

The exclusion criteria were: (1) studies examining type 1 or type 2 diabetes as the outcome; (2) fruit and vegetable juice consumption as the exposure; (3) clinical trials, reviews, case reports, or animal studies; (4) duplicate data from the same study population; and (5) non-English publications. When multiple articles originated from the same study, the most comprehensive report was included.

### 2.3. Data Extraction

Studies meeting the eligibility criteria were included in the data extraction. Two independent authors extracted the following information: first author, year of publication, country of the participants, outcome assessment, number of participants (prediabetes/normal glucose tolerance [NGT]), age range (years), dietary assessment method, definition of fruit and vegetable consumption, exposure categories (highest versus lowest), OR with 95% CI, and variables adjusted for in the analyses.

### 2.4. Main and Subgroup Analyses

The primary analysis evaluated the association between fruit and vegetable consumption and prediabetes risk using adjusted ORs with 95% CIs. In each study, the group with the highest level of fruit and vegetable consumption was compared with the group with the lowest level. Subgroup analyses were conducted according to study design, sex, age, dietary patterns, definition of prediabetes, outcome assessment methods, geographic region, and methodological quality.

### 2.5. Quality Assessment Using Risk of Bias

Risk of bias was evaluated using the Newcastle-Ottawa Scale (NOS) [20] for cross-sectional, case–control, and cohort studies. Each study was assessed across three domains: selection (maximum 3 points), comparability (maximum 2 points), and exposure (maximum 3 points). Total NOS scores ranged from 0 to 9, with higher scores indicating lower risk of bias. Studies with scores ≥ 7 were classified as high quality, scores of 4–6 as moderate quality, and scores ≤ 3 as low quality. Seven to nine scores were considered “Low risk of bias”, four to six scores were considered “Unclear risk of bias” and three or less scores were considered “High risk of bias”

### 2.6. Statistical Analysis

Adjusted ORs and 95% CIs reported in individual studies were used to estimate pooled ORs comparing the highest versus lowest (reference) exposure categories. The pooled effect estimate was calculated using the inverse variance weighted mean of the logarithm of adjusted ORs with 95% CIs.

Heterogeneity among studies was assessed using the Higgins *I*^2^ statistic, which quantifies the proportion of total variation attributable to between-study heterogeneity [21]. *I*^2^ values range from 0% (no observed heterogeneity) to 100% (maximal heterogeneity), with values >50% indicating substantial heterogeneity. A random-effects meta-analysis based on the DerSimonian–Laird method was applied because the included studies differed in design and population characteristics [22].

Publication bias was evaluated using Begg’s funnel plot and Egger’s test. Publication bias was considered present when the funnel plot showed asymmetry or when the *p*-value from Egger’s test was <0.05. All statistical analyses were performed using Stata SE version 16 (StataCorp, College Station, TX, USA).

## 3. Results

### 3.1. Selection of Relevant Studies

**Figure 1:**
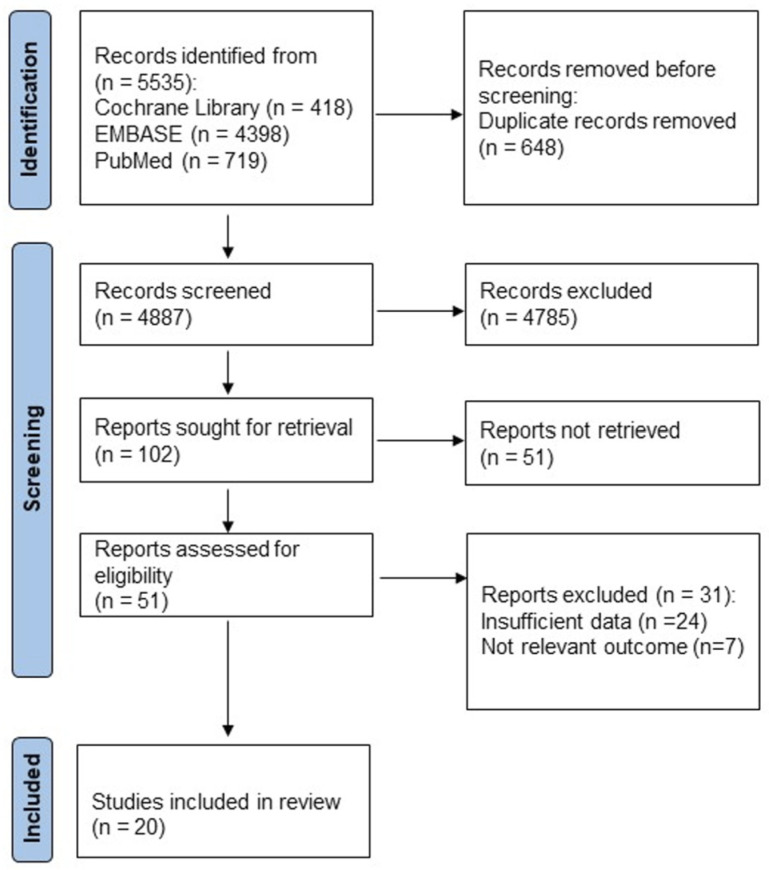
PRISMA 2020 flow chart of the article selection. PRISMA, Preferred Reporting Items for Systematic Review and Meta-Analysis.

The initial database search identified 5535 records (Figure 1). After removal of 648 duplicates, 4785 studies were excluded during title and abstract screening because they did not meet the eligibility criteria. The remaining 102 studies underwent full-text review, and 82 were excluded for the reasons presented in Figure 1. Ultimately, 20 studies [23,24,25,26,27,28,29,30,31,32,33,34,35,36,37,38,39,40,41,42] met the inclusion criteria and were included in the final meta-analysis.

### 3.2. Characteristics of Included Studies

The characteristics of the included studies are summarized in Table 1. Overall, the 20 observational studies included 24,660 cases of prediabetes among 182,387 participants. The studies comprised 16 cross-sectional studies [23,24,25,27,28,29,30,31,32,33,34,35,37,38,39,41], one case–control study [26], and three cohort studies [36,40,42].

Four studies included participants aged ≥40 years [27,34,39,42], whereas five studies included participants aged ≤65 years [26,29,33,36,41]. Regarding exposure definitions, 10 studies evaluated fruit and vegetable consumption [24,25,26,28,29,31,33,35,36,37], five evaluated vegetable consumption alone [27,30,34,40,41], four evaluated adherence to the Mediterranean dietary pattern [23,32,38,39], and one evaluated fruit consumption alone [42].

Geographically, 10 studies were conducted in Asia [25,28,29,33,34,35,37,40,41,42], seven in Europe [23,24,27,30,32,36,39], and one in America [38].

### 3.3. Methodological Quality

Table 2 presents the methodological quality of the included studies assessed using the NOS. All studies had scores > 4. The mean NOS score was 6.9 for cross-sectional and case–control studies and 7.7 for cohort studies. Studies with scores ≥ 7 were classified as high quality, whereas those with scores of 4–6 were considered moderate quality.

Based on these criteria, 14 studies were categorized as high quality, including 11 cross-sectional or case–control studies and three cohort studies. The remaining six studies, all cross-sectional or case–control designs, were classified as moderate quality of the included studies are summarized in Table 1. 

### 3.4. Result of the Meta-Analysis

**Figure 2:**
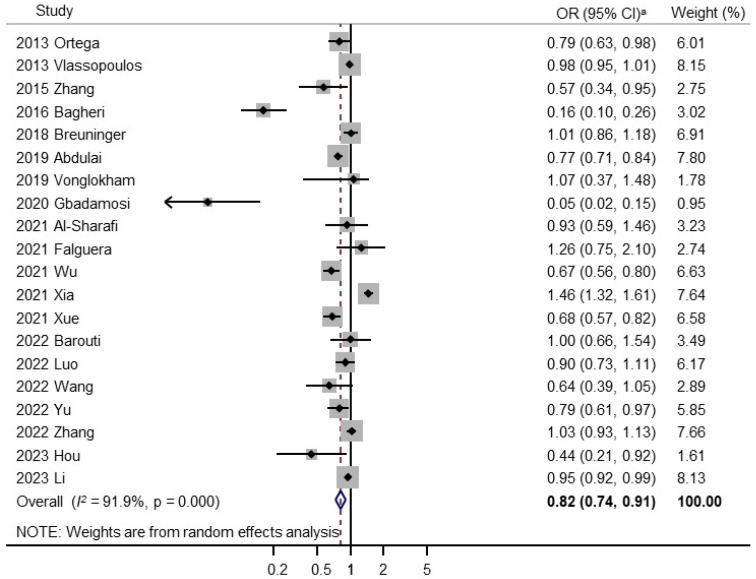
Forest plot of meta-analysis of studies on fruits and vegetables consumption and prediabetes. ^a^ Random-Effects Model. OR, Odds Ratio; CI, Confidence Interval [23,24,25,26,27,28,29,30,31,32,33,34,35,36,37,38,39,40,41,42].

Figure 2 presents the pooled association between fruit and vegetable consumption (highest versus lowest categories) and the risk of prediabetes based on a random-effects meta-analysis of the 20 observational studies. Higher fruit and vegetable consumption was significantly associated with a reduced risk of prediabetes (OR, 0.82; 95% CI, 0.74–0.91; *I*^2^ = 91.9%). The pooled analysis revealed high heterogeneity. A leave-one-out analysis with the exclusion of one study by Xia et al. [34] reduced heterogeneity (OR, 0.79; 95% CI, 0.72 to 0.87; *I*^2^ = 88.8%; n = 19) (Supplementary Figure S1). Exclusion of two studies reduced the heterogeneity to 84.3% (Supplementary Figure S2), and three studies reduced it to 78.8% (Supplementary Figure S3). The exclusion of these studies from the pooled estimate did not affect the strength of the association.

**Figure 3:**
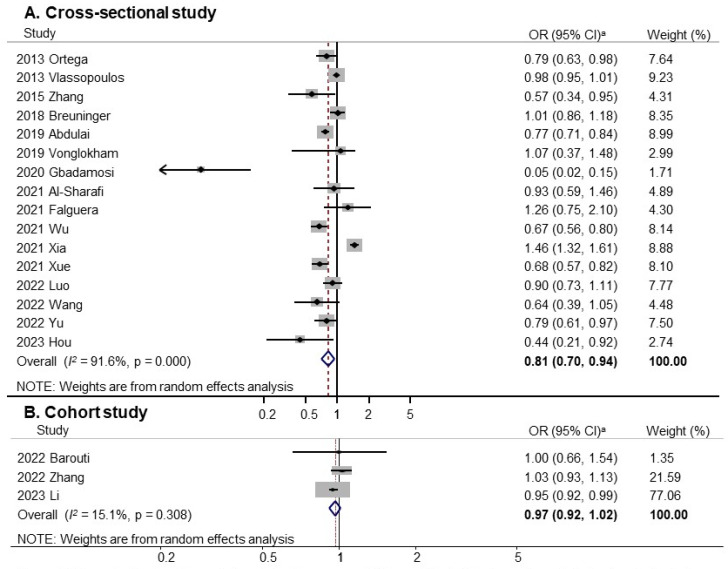
Forest plot of meta-analysis of studies on fruits and vegetables consumption and prediabetes by study design. ^a^ Random-Effects Model. OR, Odds Ratio; CI, Confidence Interval [23,24,25,26,27,28,29,30,31,32,33,34,35,36,37,38,39,40,41,42].

Subgroup analysis by study design is shown in Figure 3. A significant inverse association was observed in cross-sectional studies (OR, 0.81; 95% CI, 0.70–0.94; *I*^2^ = 91.6%; n = 16), whereas no significant association was found in cohort studies (OR, 0.97; 95% CI, 0.92–1.02; *I*^2^ = 15.1%; n = 3).

**Figure 4:**
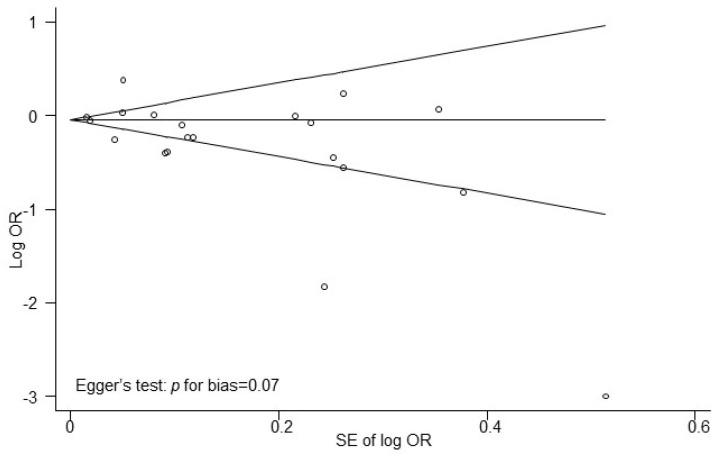
Funnel plots for identifying publication bias in the meta-analysis of observational studies.

Publication bias was assessed using Begg’s funnel plot and Egger’s test. The funnel plot appeared symmetric (Figure 4), and Egger’s test indicated no evidence of publication bias among the 20 studies (*p* for bias = 0.07).

### 3.5. Subgroup Meta-Analyses

Subgroup meta-analyses were conducted to evaluate the potential influence of sex, age, dietary patterns, definition of prediabetes, outcome assessment methods, geographic region, and methodological quality. The results are presented in Table 3.

In analyses stratified by sex, fruit and vegetable consumption was significantly associated with a reduced risk of prediabetes among male participants (OR, 0.77; 95% CI, 0.61–0.97; *I*^2^ = 63.3%; n = 4), but no significant association was observed among female participants (OR, 0.97; 95% CI, 0.69–1.37; *I*^2^ = 82.1%; n = 3).

In subgroup analyses by dietary pattern, both fruit and vegetable consumption (OR, 0.72; 95% CI, 0.60–0.87; *I*^2^ = 91.9%; n = 10), fruit consumption alone (OR, 0.82; 95% CI, 0.71–0.94; *I*^2^ = 93.1%; n = 9), and adherence to the Mediterranean diet (OR, 0.77; 95% CI, 0.60–1.00; *I*^2^ = 54.4%; n = 5) were significantly associated with reduced prediabetes risk. However, no significant association was observed for vegetable consumption alone (OR, 0.86; 95% CI, 0.69–1.07; *I*^2^ = 94.4%; n = 8).

Subgroup analyses according to prediabetes assessment showed significant associations for impaired fasting glucose (OR, 0.75; 95% CI, 0.66–0.85; *I*^2^ = 88.3%; n = 18), impaired glucose tolerance (OR, 0.74; 95% CI, 0.61–0.89; *I*^2^ = 90.5%; n = 10), and HbA1c (OR, 0.84; 95% CI, 0.71–1.00; *I*^2^ = 87.0%; n = 6).

## 4. Discussion

To our knowledge, this study is the first meta-analysis to evaluate the association between fruit and vegetable consumption and prediabetes using evidence from observational studies. In addition, subgroup analyses were performed according to study design, sex, age group, and dietary patterns related to fruit and vegetable consumption. The findings suggest a potential inverse association between fruit and vegetable consumption and prediabetes; however, this observation is largely driven by cross-sectional evidence.

Fruits and vegetables are widely recognized as beneficial dietary components because they provide vitamins, dietary fiber, and diverse bioactive compounds. Despite these benefits, rapid changes in dietary patterns have resulted in fruit and vegetable consumption that remains below recommended levels in many populations [43,44,45]. Misconceptions regarding dietary management may contribute to this pattern. For example, some individuals with diabetes believe that sweet foods or foods containing natural sugars should be avoided because they may increase blood glucose levels. Such beliefs may lead to reduced fruit consumption despite its potential metabolic benefits [46].

Numerous studies have examined the relationship between fruit and vegetable consumption and glycemic outcomes; however, the association with type 2 diabetes risk remains inconsistent. A dose–response meta-analysis of prospective studies reported that each additional 200 g/day of fruit and vegetable consumption was associated with a relative risk of 0.98 (95% CI 0.95–1.01) for type 2 diabetes, although the associations varied according to the specific types consumed [47]. Similarly, another study found that higher consumption of fruits or green leafy vegetables was associated with a lower risk of type 2 diabetes, whereas combined increases in fruit and vegetable consumption did not show a significant effect [48].

In contrast, other evidence suggests potentially adverse associations for certain forms of consumption. One study reported that each additional serving of fruit juice was associated with a 1.05-fold increase in the risk of type 2 diabetes [49]. Meta-analyses of prospective studies have also indicated that consumption of 100% fruit juice does not provide protective effects against type 2 diabetes [50,51]. Nevertheless, a recent umbrella review reported that higher fruit and vegetable consumption was associated with a reduced risk of type 2 diabetes overall [11]. To date, no meta-analysis has comprehensively evaluated the association between fruit and vegetable consumption and prediabetes. The variability observed across previous studies suggests that the relationship between fruit and vegetable consumption and glucose metabolism may depend on factors such as food type, preparation method, form of consumption, and broader dietary patterns.

The subgroup findings of the present study require careful interpretation. First, an inverse association was observed in cross-sectional studies but not in cohort studies. This difference may reflect the limited number of cohort studies available, with only three included in the analysis, which may have reduced statistical power to detect an association.

Second, the inverse association was significant only among males. Previous studies have reported sex-related differences in the associations between dietary factors and metabolic outcomes [33,52,53], although the underlying mechanisms remain uncertain. Several explanations have been proposed. Females generally exhibit greater insulin sensitivity, whereas males often have higher visceral adiposity, which promotes free fatty acid release and contributes to insulin resistance [54]. These metabolic differences may modify the effects of dietary factors on glucose metabolism. In addition, dietary preferences and food choices may differ by sex, including preferences for specific types of vegetables, which may influence the association between vegetable consumption and diabetes risk [55]. These biological and behavioral factors may partly explain the observed sex-specific differences.

Third, substantial heterogeneity was observed across the included studies (*I*^2^ = 91.9%). This high level of heterogeneity may reflect differences in geographic regions, dietary assessment methods, definitions of exposure, and diagnostic criteria for prediabetes, as well as other methodological variations among studies. To examine the influence of individual studies on the overall heterogeneity, we conducted leave-one-out sensitivity analyses. When Xia et al. [34], Bagheri et al. [26], and Gbadamosi and Tlou [30] were sequentially excluded, the heterogeneity decreased slightly; however, the overall pooled estimate remained statistically significant and the magnitude of the association changed only minimally. These findings suggest that the observed heterogeneity is unlikely to be driven by a single influential study, but rather reflects methodological differences across studies. Therefore, the association remained statistically similar across sensitivity analyses; however, the substantial heterogeneity limits the robustness and interpretability of the findings. Nevertheless, the remaining high heterogeneity warrants cautious interpretation of the results, and future studies using more standardized dietary assessment methods and consistent diagnostic criteria for prediabetes are needed to provide more comparable evidence. In addition, some subgroup analyses were based on a limited number of studies, which may have reduced statistical power to detect differences and limited the ability to fully explain the sources of heterogeneity. Therefore, these subgroup findings should be interpreted with caution.

Fourth, most of the studies included in this meta-analysis were cross-sectional studies, whereas only three were prospective cohort studies. The subgroup analysis also showed that the significant association between fruit and vegetable consumption and the risk of prediabetes was mainly observed in cross-sectional studies, while no statistically significant association was found in cohort studies. Because cross-sectional studies assess exposure and outcome at the same time point, they cannot establish temporal relationships and may be susceptible to reverse causation, in which individuals with existing health conditions may have already modified their dietary habits. Therefore, the observed association should not be interpreted as evidence of a causal relationship. Given that only a limited number of cohort studies have examined the relationship between fruit and vegetable consumption and the risk of prediabetes, future meta-analyses including a larger number of prospective cohort studies may provide clearer evidence regarding this association.

Fifth, the association with prediabetes appeared stronger for fruit consumption alone or combined fruit and vegetable consumption than for vegetable consumption alone. Previous research has also suggested that fruit consumption shows a more consistent inverse association with diabetes risk than vegetable consumption, with certain fruit subtypes contributing more strongly to this relationship [47]. Furthermore, the form of vegetable consumption may influence metabolic outcomes. Some studies have reported that raw vegetable consumption is more strongly associated with reduced diabetes risk, potentially because of higher dietary fiber intake and lower glycemic index of meals [56]. Because the nutritional composition and glycemic responses of vegetables can vary substantially depending on preparation methods, simple measures of total vegetable consumption may not fully capture their health effects.

This study has several limitations. First, several included studies used cross-sectional designs, which may introduce recall bias in dietary assessment and limit causal inference. Second, adjustment for lifestyle factors, including physical activity, socioeconomic status, and overall diet quality, varied across studies; therefore, residual confounding cannot be excluded. Third, because relatively few studies examined fruit and vegetable consumption in relation to prediabetes, detailed analyses according to specific dietary factors—such as consumption forms, food types, and preparation methods—were not possible. In addition, most of the included studies assessed dietary intake using self-reported methods such as food frequency questionnaires (FFQs) or dietary recalls. These methods are subject to measurement error and may lead to exposure misclassification. Such limitations in dietary assessment may have influenced the estimated association between fruit and vegetable consumption and the risk of prediabetes. In the absence of a formal certainty assessment framework, we have interpreted the findings by considering study design, risk of bias, consistency, and heterogeneity across studies.

Despite these limitations, this study has several strengths. To our knowledge, it is the first meta-analysis to comprehensively evaluate the association between fruit and vegetable consumption and prediabetes. Subgroup analyses were conducted according to study design, sex, age group, consumption patterns, diagnostic criteria for prediabetes, and geographic region, enabling a systematic evaluation of consistency and heterogeneity across studies. Importantly, the findings suggest that fruit and vegetable consumption may be a relevant dietary factor; however, current evidence is insufficient to establish a clear or causal relationship with prediabetes. These findings may contribute to the existing body of literature; however, further well-designed prospective studies are required before drawing firm conclusions for dietary recommendations.

## 5. Conclusions

This meta-analysis demonstrated that fruit and vegetable consumption may be associated with beneficial effects. As the first meta-analysis examining this relationship in observational studies, the findings indicate that fruit and vegetable consumption may play a beneficial role during the early stages of glucose dysregulation. However, the available evidence remains limited, particularly regarding prospective cohort studies and detailed dietary variables such as food types, preparation methods, and consumption patterns. Therefore, additional well-designed prospective cohort studies are required to clarify the association between fruit and vegetable consumption and prediabetes and to better understand the role of specific dietary components in preventing progression to type 2 diabetes.

## Figures and Tables

**Table 1:** General characteristics of the studies included in the final analysis.

Study	Country, (Study Name)	Outcome Assessment	Participants (Prediabetes/NGT)	Age Range (Years)	Dietary Assessment	Definition of Fruit and Vegetable Consumption	Categories of Exposure (Highest vs. Lowest Category)	OR (95%CI)	Adjusted Variables
Cross-sectional study									
2013 Ortega [23]	Spain (The Di@bet.es Study)	Impaired glucose tolerance (IGT) and/or impaired fasting glucose (IFG) according to the 1999 WHO criteria	4598 (826/3772)	≥18	FFQ. Interviewer administered	MedDiet score	High (>26) vs. low (<23)	0.79 (0.63–0.98)	Age, BMI and WC, sex, educational level, civil status, hypertension, dyslipidemia, physical exercise, smoking status, family history of diabetes in 1st-degree relatives
2013 Vlassopoulos [24]	United Kingdom (The Scottish Health Surveys)	Glycated hemoglobin (HbA1c) between 5.7% (39 mmol/mol) and 6.4% (46 mmol/mol)	5546 (1391/4155)	18–95	24 h dietary recall. Interviewer administered	Fruit and vegetable	Yes vs. no	0.98 (0.95–1.01)	Age, sex, ethnic group, social class, physical activity level, CRP, BMI, waist circumference, year of study, smoking status
2015 Zhang [25]	China	Fasting plasma glucose (FPG) concentration of 110–126 mg/dL (6.1–7.0 mmol/L)	1459 (132/1327)	20–75	One-month dietary recall. Interviewer administered	Vegetable–fruit pattern	Highest tertile (T3) vs. lowest tertile (T1)	0.57 (0.34–0.95)	Age, BMI, total energy intake, drinking status, smoking status, physical activity status
2018 Breuninger [27]	Germany (Cooperative Health Research in the Region of Augsburg (KORA) FF4 study)	IFG (5.6–6.9 mmol/L fasting glucose), IGT (7.8–11.0 mmol/L 2 h glucose) or the combination of both according to the 2003 ADA criteria	1334 (545/789)	46–71	24 h dietary recall and FFQ. Self-administered	Vegetables	One standard deviation vs. habitual dietary intake	1.01 (0.86–1.18)	Age, sex, energy intake, BMI, waist circumference, family history of diabetes, physical activity, smoking, education, hypertention
2019 Abdulai [28]	China	IFG (6.1–6.99 mmol/L) according to the WHO criteria	35,410 (2670/32,740)	≥20	FFQ. Interviewer administered	Fruits/vegetables	Yes vs. no	0.77 (0.71–0.84)	Age, gender, smoking, drinking, exercise, hypertension, family history of diabetes, waist circumference, BMI, metabolic syndrome, dyslipidaemia
2019 Vonglokham [29]	Laos	FPG levels 6.1 to <7.0 mmol/L	2347 (56/2291)	18–64	FFQ. Interviewer administered	Fruit and vegetable	>5 servings/d vs. no	1.07 (0.37–1.48)	Age, sex, education, marital status, ethno-linguistic group, urban residence, BMI, central obesity, physical activity, high sitting time, current tobacco, drinking, hypertensive, raised cholesterol
2020 Gbadamosi [30]	Swaziland	IFG and IGT were defined according to the WHO criteria	357 (25/332)	≥18	FFQ. Self-administered	Vegetables	≥3 servings/d vs. no	0.05 (0.02–0.15)	Smoking, consumption of salty processed foods, consumption of fruits
2021 AI-Sharafi [31]	Yemen	IFG 100–125 mg/dL, and oral glucose tolerance test (OGTT) (75 g glucose 2 h) or HbA1c 5.7–6.4% according to the ADA criteria	547 (208/339)	20–70	FFQ. Interviewer administered	Fruit and vegetable	Yes vs. no	0.93 (0.59–1.46)	None
2021 Falguera [32]	Spain (The Mollerussa Prospective Observational Cohort Study)	FPG from 100 mg/dL to <126 mg/dL or HbA1c from 5.7% to <6.5%	535 (216/319)	≥25	FFQ, Interviewer administered	aMED score	Highest tertile (5–8) vs. lowest tertile (0–3)	1.26 (0.75–2.10)	Age, sex, BMI, education level, hypertension, dyslipidemia, physical activity
2021 Wu [33]	China (The China Health and Nutrition Survey (CHNS))	FPG from ≥6.1 to <7.0 mmol/L or HbAlc from ≥5.7% to <6.5% (Association AD 2018; Chinese Diabetes Society 2018)	6228 (1980/4248)	18–65	24 h dietary recall. Interviewer administered	Fruit and vegetable	Highest quintile (≥533.3 g/d) vs. lowest quintile (<229.8 g/d)	0.67 (0.56–0.80)	Age, residence, education level, smoking status, alcohol intake, history of hypertension, daily energy intake, leisure physical activity, BMI, red meat intake, whole grains intake, legumes intake
2021 Xia [34]	China	Defined following the WHO criteria	45,892 (1643/44,249)	45–101	FFQ. Interviewer administered	Vegetable	≥100 g/d vs. <100 g/d	1.46 (1.32–1.61)	Age, sex, education, income, comorbidities, smoking, drinking, sleep duration, family history of diabetes, BMI, abdominal obesity
2021 Xue [35]	China	5.6 mmol/L ≤ FPG < 7.0 mmol/L or 5.7% < HbA1c < 6.5% according to the 2018 ADA criteria	35,125 (2634/32,491)	18–79	FFQ. Interviewer administered	Vegetable–fruit pattern	Highest quintile (Q5) vs. lowest quintile (Q1)	0.68 (0.57–0.82)	Age, region, gender, education level, marital status, per capita monthly income, BMI, smoking, alcohol drinking, physical activity, family history of diabetes, energy
2022 Luo [37]	China (The Qingdao Diabetes Prevention Program)	5.6 mmol/L ≤ FPG < 7.0 mmol/L or 7.8 mmol/L ≤ 2 h PG < 11.1 mmol/L according to the 2018 ADA criteria	3681 (1330/2351)	35–74	FFQ. Interviewer administered	Fruit–vegetable pattern	Highest quartile (Q4) vs. lowest quartile (Q1)	0.90 (0.73–1.11)	Age, sex, educational attainment, marital status, urban-rural distribution, personal monthly income, family history of diabetes, occupational physical activity, smoking, hypertension, BMI, TG, total energy intake
2022 Wang [38]	USA (Hispanic Community Health Study-Study of Latino)	FPG ≥ 100 mg/dL (5.5 mmol/L) and <126 mg/dL (7 mmol/L); a 2 h post-load glucose level (2 hPG) ≥ 140 mg/dL (7.7 mmol/L) and <200 mg/dL (11.2 mmol/L); A1c level ≥ 5.7% (39 mmol/mol) and <6.5% according to the ADA criteria	1199 (805/394)	23–83	24 h dietary recall. Interviewer administered	MedDiet index	Highest tertile (mean 36.2) vs. lowest tertile (mean 27.4)	0.64 (0.39–1.05)	Age, sex, total energy intake, physical activity, metformin use, antibiotic use, probiotic use, place of birth, age at relocation to the us mainland, Bristol stool scale, BMI
2022 Yu [39]	Netherland (The Maastricht Study)	FPG of 6.1–6.9 mmol/L and no hypoglycaemic medications (GMS score = 1) according to the 2006 WHO criteria	2474 (514/1960)	40–75	FFQ. Self-administered	MED score	Highest tertile (6–9) vs. lowest tertile (0–3)	0.79 (0.61–0.97)	Age, sex, BMI, level of education, level of household income, smoking status, daily energy intake, daily glucose intake, estimated glomerular filtration rate, total physical activity, usage of lipid-modification medication, history of cardiovascular disease, the year for metabolomics measurement
2023 Hou [41]	Taiwan	Abnormal fasting glucose referred to 100–125 mg/dL and abnormal glycated hemoglobin referred to 5.7–6.4% according to the ADA criteria	242 (147/95)	20–65	24 h dietary recall. Interviewer administered	Vegetables	≥2 servings/d vs. none	0.44 (0.21–0.92)	Age, gender, BMI
Case–control study									
2016 Bagheri [26]	Iran	Fasting blood glucose (FBG) between 5.6 and 6.9 mmol/L or OGTT 7.8–11 mmol/L	300 (150/150)	35–65	FFQ. Interviewer administered	VFL dietary pattern	Highest tertile (T3) vs. lowest tertile (T1)	0.16 (0.10–0.26)	Age, education, physical activity, BMI, energy intake
Cohort study									
2022 Barouti [36]	Sweden (The Stockholm Diabetes Prevention Program (SDPP))	IFG 6.1–6.9 and 2 h glucose < 7.8 mmol/L; IGT < 6.1 and 2 h glucose 7.8–11.0 mmol/L according to the 1999 WHO criteria	5997 (870/5127)	35–65	FFQ. Self-administered	Total fruit and vegetable	Highest tertile (T3) vs. lowest tertile (T1)	1.00 (0.66–1.54)	Age, family history of diabetes, education, socioeconomic index group, high blood pressure, physical activity, smoking, alcohol, wholegrain intake, yoghurt/sour milk intake, BMI
2022 Zhang [40]	China (The Tianjin Chronic Low-grade Systemic Inflammation and Health (TCLSIH) Cohort Study)	FPG of 5.6–6.9 mmol/L, and/or 2 h plasma glucose of 7.8–11.0 mmol/L in the oral glucose tolerance test, and/or an HbA1c of 5.7–6.4% (36–46 mmol/mol) without a history of diabetes according to the ADA criteria	18,085 (4139/13,946)	18–90	FFQ. Self-administered	Vegetable fiber	Highest quartile (Q4) vs. lowest quartile (Q1)	1.03 (0.93–1.13)	Age, sex, baseline BMI, smoking status, alcohol drinking status, educational level, occupation, household income per month, physical activity, metabolic syndrome, family history of disease, long-term use of medications, total energy intake, total protein intake, total fat intake, added sugar intake, intake of the other fiber sources
2023 Li [42]	China (The China Cardiometabolic Disease and Cancer Cohort Study)	Either IFG (FPG 5.6–6.9 mmol/L, and OGTT 2 hPG < 7.8 mmol/L), IGT (FPG < 7.0 mmol/L and OGTT 2 hPG 7.8–11.0 mmol/L) or combined IFG/IGT	21,031 (4379/16,652)	≥40	FFQ. Interviewer administered	100 g/d intake of fresh fruit	Yes vs. no	0.95 (0.92–0.99)	Age, sex, BMI, waist circumference, physical activity, sedentary time, smoking and drinking status, education level, family history of diabetes, triglycerides, LDL-C, HDL-C

Abbreviations: NGT, normal glucose tolerance; OR, Odds Ratio; CI, Confidence Interval; MedDiet, Mediterranean diet; IGT, impaired glucose tolerance; IFG, impaired fasting glucose; BMI, body mass index; HbA1c, Glycated hemoglobin A1c; CRP, serum C-reactive protein; FPG, fasting plasma glucose; ADA, American Diabetes Association; WHO, World Health Organization; OGTT, oral glucose tolerance test; aMED, alternative Mediterranean Diet score; TG, triglyceride; GMS, glucose metabolism status; MED, Mediterranean diet; FBG, fasting blood glucose; VFL, vegetables, fruits and legumes; LDL-C, low-density lipoprotein cholesterol; HDL-C, high-density lipoprotein cholesterol.

**Table 2:** Methodological quality of the included studies based on the Newcastle-Ottawa Scale.

**Cross-Sectional and Case–Control Study (n = 17)**	**Selection**				**Comparability**		**Exposure**			**Total**
	**1**	**2**	**3**	**4**	**5A**	**5B**	**6**	**7**	**8**	
2013 Ortega [23]	1	1	1	0	1	1	0	1	0	6
2013 Vlassopoulos [24]	1	1	1	0	1	1	0	1	0	6
2015 Zhang [25]	1	1	1	1	1	1	0	1	0	7
2016 Bagheri [26]	1	1	1	1	1	1	0	1	1	8
2018 Breuninger [27]	1	1	1	1	1	1	0	1	1	8
2019 Abdulai [28]	1	1	1	1	1	1	0	1	0	7
2019 Vonglokham [29]	1	1	1	0	1	1	0	1	0	6
2020 Gbadamosi [30]	1	1	0	0	0	1	0	1	0	4
2021 AI-Sharafi [31]	1	1	1	1	0	0	0	1	0	5
2021 Falguera [32]	1	1	1	1	1	1	0	1	1	8
2021 Wu [33]	1	1	1	1	1	1	0	1	1	8
2021 Xia [34]	1	1	1	1	1	1	0	1	1	8
2021 Xue [35]	1	1	1	1	1	1	0	1	1	8
2022 Luo [37]	1	1	1	1	1	1	0	1	1	8
2022 Wang [38]	1	1	1	0	1	1	0	1	0	6
2022 Yu [39]	1	1	1	0	1	1	0	1	1	7
2023 Hou [41]	1	1	0	1	1	1	0	1	1	7
**Cohort study (n = 3)**	**Selection**				**Comparability**		**Exposure**			
	**1**	**2**	**3**	**4**	**5A**	**5B**	**6**	**7**	**8**	
2022 Barouti [36]	1	1	0	1	1	1	1	1	0	7
2022 Zhang [40]	1	1	0	1	1	1	1	1	1	8
2023 Li [42]	1	1	1	1	1	1	1	0	1	8

Newcastle-Ottawa Scale (NOS) for cross-sectional and case–control studies (Yes = 1, No = 0). (1) Adequate definition of cases. (2) Cases are consecutive or obviously representative. (3) Selection of controls. (4) Definition of controls. (5A) Comparability of cases and controls on the basis of the design or analysis adjusted for age. (5B) Comparability of cases and controls on the basis of the design or analysis adjusted for additional factor. (6) Ascertainment of expose. (7) Same method of ascertainment for participants. (8) Non-response rate. Newcastle-Ottawa Scale (NOS) for cohort studies (Yes = 1, No = 0). (1) Representativeness of the expose cohort. (2) Selection of the non-exposed cohort. (3) Ascertainment of expose. (4) Demonstration that outcome was not present at start of study. (5A) Comparability of cases and controls on the basis of the design or analysis adjusted for age. (5B) Comparability of cases and controls on the basis of the design or analysis adjusted for additional factor. (6) Ascertainment of outcome. (7) Follow-up long enough for outcome to occur. (8) Adequacy of follow-up of cohort.

**Table 3:** Subgroup meta-analyses of studies on fruit and vegetable consumption and prediabetes.

Factors	Number of Studies	Summary OR (95% CI)	Heterogeneity, *I*^2^ (%)
Study design			
Cross-sectional study	16	0.81 (0.70–0.94)	91.6
Cohort study	3	0.97 (0.92–1.02)	15.1
Case–control study	1	0.16 (0.10–0.26)	0.0
Sex			
Men	4	0.77 (0.61–0.97)	63.3
Women	3	0.97 (0.69–1.37)	82.1
Age			
65 years and younger	5	0.55 (0.30–1.01)	89.9
40 years and older	4	1.04 (0.81–1.33)	95.5
Dietary patterns			
Vegetable	8	0.86 (0.69–1.07)	94.4
Fruit	9	0.82 (0.71–0.94)	93.1
Fruit and vegetable	10	0.72 (0.60–0.87)	91.9
Mediterranean diet	5	0.77 (0.60–1.00)	54.4
Fruits-vegetables pattern	4	0.50 (0.29–0.87)	92.9
Definition of prediabetes			
ADA	7	0.85 (0.72–1.01)	73.8
WHO	6	0.73 (0.50–1.07)	96.3
Assessment of outcome			
IFG	18	0.75 (0.66–0.85)	88.3
IGT	10	0.74 (0.61–0.89)	90.5
HbA1c	6	0.84 (0.71–1.00)	87.0
Region			
America	1	0.64 (0.39–1.05)	0.0
Asia	10	0.86 (0.73–1.01)	93.3
Europe	7	0.84 (0.68–1.04)	85.5
Methodological quality			
High quality	14	0.80 (0.69–0.93)	92.8
Moderate quality	6	0.68 (0.48–0.98)	87.4

Abbreviations: OR, Odds Ratio; CI, Confidence Interval; ADA, American Diabetes Association; WHO, World Health Organization; IFG, impaired fasting glucose; IGT, impaired glucose tolerance; HbA1c, Glycated hemoglobin A1c.

## Data Availability

These data were derived from the following resources available in the public domain: [Korea National Health and Nutrition Examination Survey (KNHANES)] [https://knhanes.kdca.go.kr].

## Supplementary Materials

The following supporting information can be downloaded at: https://www.mdpi.com/article/10.3390/nu18091391/s1, Supplementary Figure S1. Forest plot of meta-analysis of studies on fruit and vegetable consumption and prediabetes after exclusion study of Xia et al. 2021 [34]. Supplementary Figure S2. Forest plot of meta-analysis of studies on fruit and vegetable consumption and prediabetes after exclusion study of Bagheri et al. 2016 [26] and Xia et al. 2021 [34]. Supplementary Figure S3. Forest plot of meta-analysis of studies on fruit and vegetable consumption and prediabetes after exclusion study of Bagheri et al. 2016 [26], Gbadamosi and Tlou 2020 [30], and Xia et al. 2021 [34].
