# Supplementary material for: Effects of Dietary Fruit and Vegetable Consumption on Prediabetes: A Systematic Review and Meta-Analysis
Source: Nutrients. 2026 Apr 28;18(9):1391. doi: 10.3390/nu18091391 (PMC13164783; doi:10.3390/nu18091391)
Supplement: Supplementary file 1 [file nutrients-18-01391-s001.zip › nutrients-4243060-supplementary.pdf]

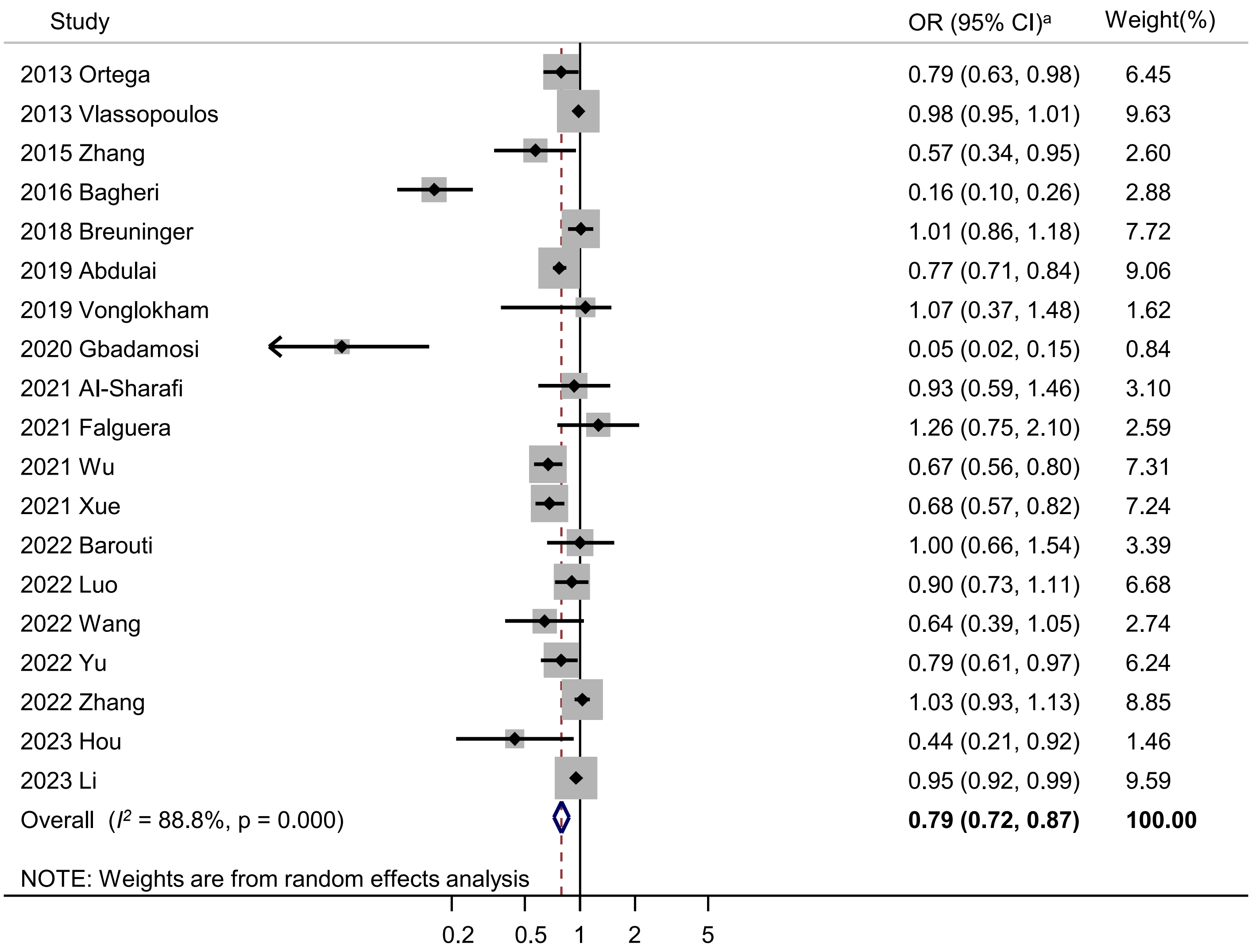

Supplementary Figure S1. Forest plot of meta-analysis of studies on fruit and vegetable consumption and prediabetes after exclusion study of Xia et al. 2021 [34]. [a] Random-Effects Model. OR, Odds Ratio; CI, Confidence Interval. [23-33,35-42]

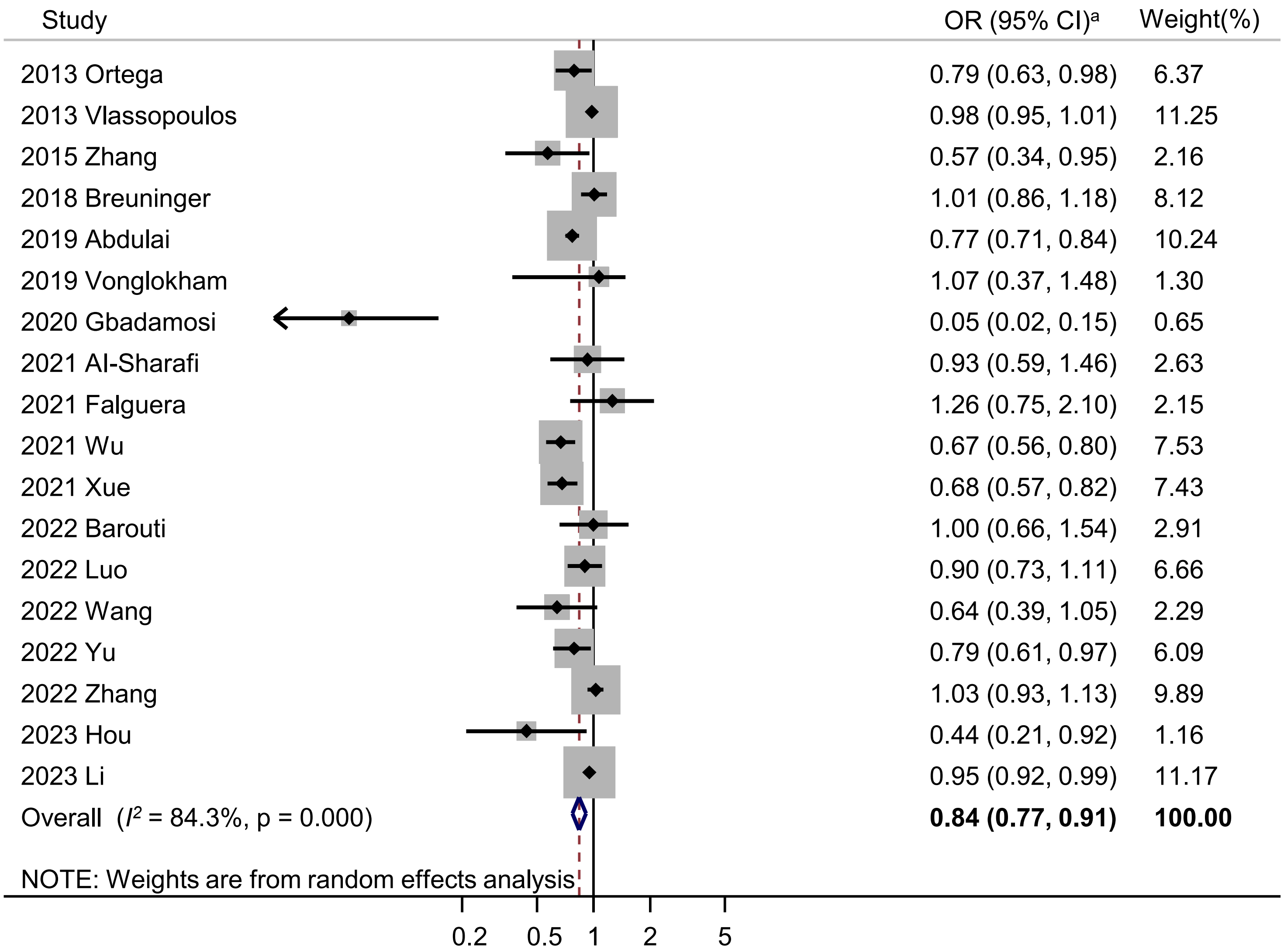

Supplementary Figure S2. Forest plot of meta-analysis of studies on fruit and vegetable consumption and prediabetes after exclusion study of Bagheri et al. 2016 [26] and Xia et al. 2021 [34]. [A] Random-Effects Model. OR, Odds Ratio; CI, Confidence Interval. [23-25,27-33,35-42]

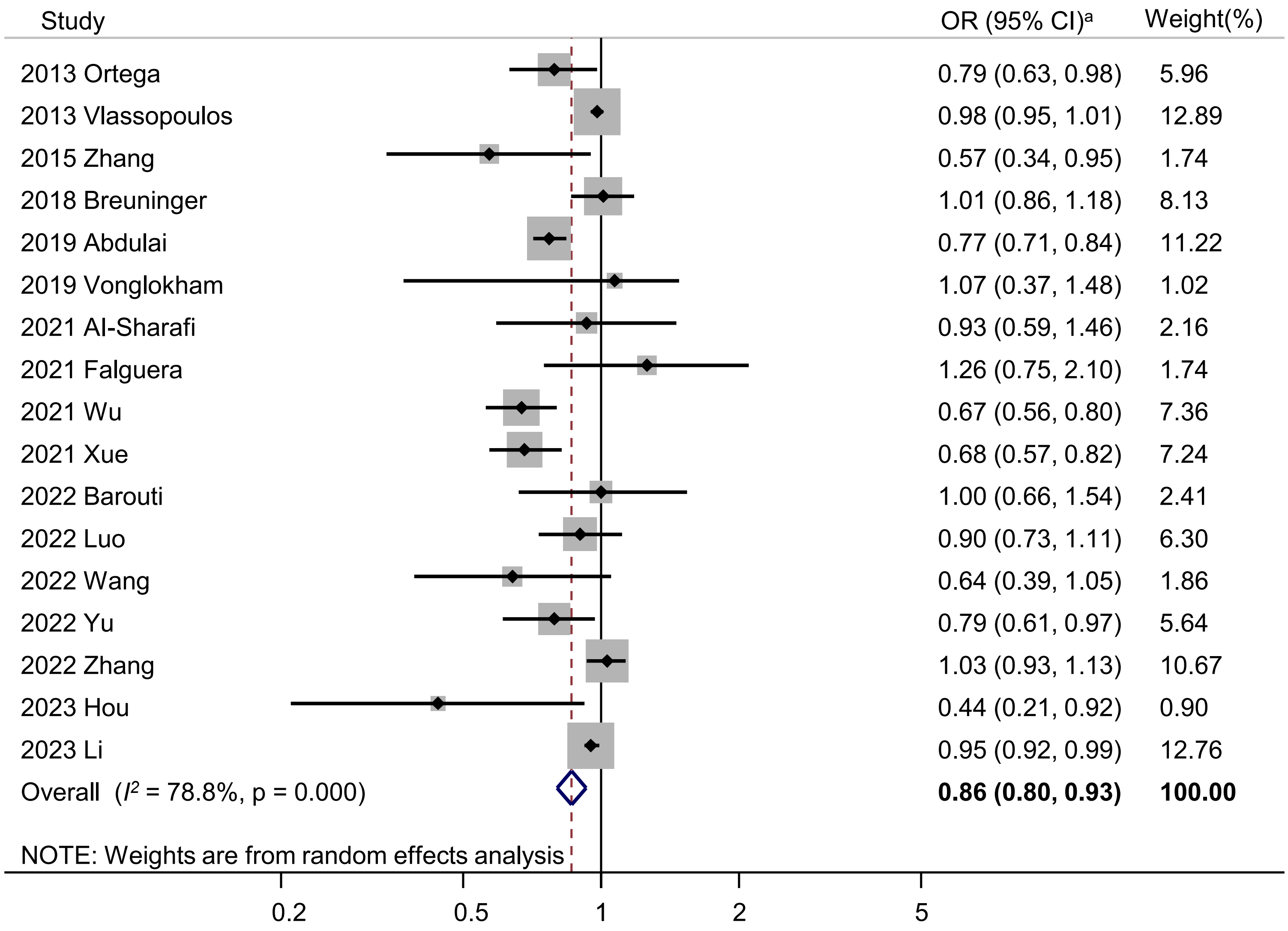

Supplementary Figure S3. Forest plot of meta-analysis of studies on fruit and vegetable consumption and prediabetes after exclusion study of Bagheri et al. 2016 [26], Gbadamosi and Tlou 2020 [30], and Xia et al. 2021 [34]. [a] Random-Effects Model. OR, Odds Ratio; CI, Confidence Interval. [23-25,27-29,31-33,35-42]
